# The Institutional Worker

**Published:** 1912-07-20

**Authors:** 


THE
### INSTITUTIONAL WORKER
**Being a Special Supplement to "The Hospital"**

### Editor's Notices.

**Contributions:**

Contributions should be written, or preferably typed, on one side of the paper only, and all articles sent in are accepted upon the distinct understanding that they are forwarded to The Hospital only.

The Editor cannot undertake to return MSS. not used, but when a stamped directed envelope is enclosed the MSS. may be returned if a special request is made.

Accepted articles and paragraphs of news will be paid for after publication at the scale rate.

**Address:**

To prevent delay all contributions and letters on editorial business must be addressed exclusively to the Editor, "The Hospital," 29 Southampton Street, Strand, London, W.C. It is important that this regulation shall be strictly observed.

**Correspondence:**

Correspondence on all subjects is invited. The name and address of correspondents must be given as a guarantee of good faith, but not necessarily for publication.

**Special Articles:**

Special articles are invited, and questions, inquiries, and paragraphs upon all matters relating to the work, administration, and management of general, special, mental, fever, cottage and convalescent hospitals, sanatoria, homes, institutions, societies and organisations for the treatment or care of the sick, injured and dependants of all classes. Every matter affecting the interests and welfare of the staff of all grades working in these institutions will receive special consideration.

**Administrative Medicine, Research and Items of News:**

Special terms will be paid for approved articles on subjects in this wide field by experts who have made some section of it their special study and interest. We wish to give prominence to every movement tending to advance accuracy of diagnosis, efficiency in treatment, and the development of modern methods to eradicate disease and promote the welfare of the suffering.

**A Bureau of Information:**

We invite inquiries and applications for information and help in providing for that numerous class of sufferers who require special care or house-room which they cannot obtain in their own homes or secure for themselves. **We cannot, however, prescribe, or recommend practitioners.**

**Books for Review:**

Publishers are particularly requested to send advance proofs of any new books of importance whenever possible, as the Editor has made arrangements to publish immediate reviews on a new plan.

**Photographs, Plans, Blocks, and Illustrations:**

It is requested that wherever possible MSS. may be accompanied by illustrations in any of the above forms. The name of the sender and of the article to which it belongs should be written on the back of each photograph, drawing, or block for purposes of identification.

**Local Papers:**

Newspapers containing reports or news paragraphs **should be marked** and addressed to the Sub-Editor.

**The Coupon System:**

A coupon will be found at the bottom of the third inside page of the cover each week. This coupon must be attached to every question or inquiry to which an answer is desired in The Hospital.

### A Want Supplied.

The many difficulties that have hitherto presented themselves to workers seeking accurate information on Hospital and Institution affairs, the prevention of diseases, the care of the sick, and alleviation of the suffering, need no longer prove practically insurmountable. The Bureau of Information instituted by The Hospital is open to every reader of the Journal, and enquiries on all subjects are cordially invited. The accumulation of many years' experience in Institutional matters and in every branch of the Medical, Health and Sanitary Services is at the disposal of anyone requiring assistance, and questions are dealt with by experts who are best able to cope with problems that are placed before the Bureau. Our readers, therefore, can rely with every assurance upon the authenticity of the information supplied. Such a departure has long been needed, and the fact that applications for information and help are being received from all parts of the country is evidence that the Conductors of The Hospital have supplied a long-felt want. All Communications should be addressed The Hospital Bureau of Information, The Hospital Building, 28 and 29 Southampton Street, Strand, London, W.C.

### Manager's Notices.

**All letters on business matters must be addressed to The Manager, "The Hospital," 28 and 29 Southampton Street, London, W.C.**

**Advertisements:**

To ensure insertion, all advertisements for The Hospital must reach the Manager not later than Wednesday morning in each week. For scale of charges see pages v and 2.

**Subscriptions,** which may commence at any time, are payable in advance. Cheques and Post-Office Orders should be crossed London County and Westminster Bank, Covent Garden Branch, and made payable to the Manager of The Hospital.

Rates of Subscription (including Postage).

| *United Kingdom.* | | *Foreign and Colonial.* | |
|---|---|---|---|
| Three Months ... ... | 2s. 0d. | Three Months ... ... | 2s. 6d. |
| Six Months ... ... | 4s. 0d. | Six Months ... ... | 5s. 0d. |
| Twelve Months ... ... | 6s. 6d. | Twelve Months ... ... | 8s. 8d. |

*SUBSCRIPTION FORM.*

*Please send me* The Hospital *for..................months,*

*commencing with your issue of.........................., for*

*which please find enclosed...........................*

*Name.....................................................*

*Address* ..................................................

..................................................

*Date.......................................*

*To* ..................................... *Newsagent.*

**Remittances:**

**It is especially requested that remittances be made by Cheque or Postal Order, and in halfpenny stamps only when the amount is under sixpence.**

### OFFICIAL APPOINTMENTS VACANT.

### Poor-Law Vacancies.

Aged Women's Attendant (£20), Paddington Guardians. Mr. H. F. Aveling, C. to G., 313-319 Harrow Road, W. July 23.

Assistant Nurse (£28), Liverpool Guardians. Mr. G. W. Coster, C. to G., Parish Offices, Liverpool. July 22.

Assistant Nurse (£30), Dartford Union. Mr. E. J. Hobbs, C. to G., Dartford. July 22.

Assistant Nurse (£24), Holbeck Union. Mr. G. Diment, C. to G., Holbeck. July 22.

Assistant Nurse (£25), Oxford Guardians. Master of the Workhouse, Oxford. July 23.

Charge Nurse (£35), Cardiff Union. Mr. A. J. Harris, C. to G., Cardiff. July 22.

Charge Nurse (£30), Bristol Guardians. Mr. J. J. Simpson, C. to G., Bristol. July 22.

Charge Nurse (£28), Tonbridge Union. Mr. N. R. Stone, C. to G., Tunbridge Wells. July 25.

First Assistant Schoolmaster (£140 to £150 according to qualifications), Liverpool Guardians. Mr. G. W. Coster, C. to G., Parish Offices, Liverpool. July 22.

CHARGE NURSE (£35), Madeley Union. Mr. A. H. Thorn-Pudsey, C. to G., Iron Bridge. July 29.

CHARGE NURSE (£35), Banbury Union. Mr. E. L. Fisher, C. to G., Banbury. July 30.

HOUSE MOTHER (£20), Romford Union. Mr. C. Bloomfield, C. to G., Romford. July 22.

INFANTS' NURSE (£25), Bucklow Union. Mr. G. Leigh, C. to G., Knutsford. July 22.

INSTRUCTOR IN DOMESTIC ECONOMY (£85), South Manchester Guardians. Mr. D. S. Bloomfield, C. to G., All Saints, Manchester. July 23.

LAUNDRESS (£25), Blean Union. Mr. J. E. Burch, C. to G., 39 Castle Street, Canterbury. July 22.

LAUNDRESS (£52), Windsor Union. Mr. J. E. Gale, C. to G., Windsor. July 22.

MALE ATTENDANT (£30), York Union. Mr. G. Sykes, C. to G., York. July 23.

MALE LUNATIC ATTENDANT (£30), Birkenhead Union. Mr. J. Carter, C. to G., Birkenhead. July 23.

MASTER'S ASSISTANT AND COOK (married couple) (£25 and £20), Morpeth Union. Mr. H. W. Sample, C. to G., Morpeth. July 22.

NURSE (£35), Poplar Guardians. Mr. G. H. Lough, C. to G., 45 Upper North Street, Poplar, E. July 23.

NURSE (£35), Sheppey Union. Mr. J. Copland, C. to G., Sheerness. July 23.

NURSE (£30), Dolgelly Union. Mr. R. G. Jones, C. to G., Dolgelly. July 26.

NURSES (2) (£25), Nantwich Union. Mr. H. G. Atkinson, C. to G., Nantwich. July 27.

NURSES (£33), Poplar Guardians. Mr. G. H. Lough, C. to G., 45 Upper North Street, Poplar, E. July 22.

PORTER (£25), Bedford Union. Mr. W. Payne, C. to G., Bedford. July 22.

PORTER AND LABOUR MASTER (£25), Rochford Union. Mr. F. Gregson, C. to G., Southend-on-Sea. July 23.

RELIEVING OFFICER, ETC. (£120 and Commission), Walsingham Union. Mr. R. S. Butcher, C. to G., Fakenham. July 27.

SUPERINTENDENT NURSE (£35), Wisbech Union. Mr. R. W. Faircloth, Acting C. to G., Wisbech. July 22.

SUPERINTENDENT NURSE (£35), Walsingham Union. Mr. R. S. Butcher, C. to G., Fakenham. July 30.

SUPERINTENDENT OF LAUNDRY (£40), Fulham Guardians. Mr. E. J. Mott, C. to G., 129 Fulham Palace Road, W. July 23.

THIRD ASSISTANT CLERK (£80), Paddington Guardians. Mr. H. F. Aveling, C. to G., 313-319 Harrow Road, W. July 22.

### Municipal Vacancies.

DISTRICT HIGHWAY SURVEYOR (£100), Newbury R.D.C. Mr. S. V. Pinniger, Clerk to the Council, Newbury. July 26.

HEALTH VISITOR (£78), Manchester T.C. Public Health Office, Manchester. July 27.

HIGHWAY SURVEYOR, INSPECTOR OF NUISANCES, ETC. (£140), Wantage U.D.C. Mr. E. B. Ormond, Clerk to the Council, Wantage. July 27.

INSPECTOR OF NUISANCES (£100), Mynyddislwyn U.D.C. Mr. T. C. Griffiths, Clerk to the Council, Mynyddislwyn. July 22.

INSPECTOR OF NUISANCES (£120), Elham R.D.C. Mr. B. C. Drake, Clerk to the Council, Hythe. July 29.

NURSE-MATRON (£45), Earsdon Joint Hospital Board. Mr. A. Whitehorn, Clerk to the Board, 60 Saville Street, North Shields. July 25.

NURSE-MATRON (£35), Hebburn U.D.C. Isolation Hospital. Mr. T. Stuart, Clerk to the Council, Hebburn. August 5.

TOWN CLERK (£450), Luton T.C. Mr. Bruce Penny, Town Clerk, Luton. July 22.

TOWN PLANNING ASSISTANT (£3 3s. weekly), Wembley U.D.C. Mr. C. R. W. Chapman, Engineer, Public Offices, Wembley. July 22.

### Miscellaneous Vacancies.

ACCOUNTANT (£200), Pontypridd and Rhondda Joint Water Board. Mr. W. P. Nicholas, Clerk to the Board, Mill Street, Pontypridd. July 22.

### Books for Institutional Workers.

| | |
|---|---|
| "Midwife's Pocket Dictionary, with revised Rules of the C.M.B." | 1s. 1d. |
| "The Nurses' Pronouncing Dictionary" | 2s. 0d. |
| "A Handbook for Nurses." (J. K. Watson, M.D.) | 5s. 4d. |
| "Art of Feeding the Invalid." | 1s. 6d. |
| "Surgical Ward Work and Nursing." | 5s. 5d. |
| "Massage Movements, including the Nauheim Exercises" | 1s. 1d. |
| "Surgical Instruments and Appliances used in Operations" (Harold Burrows, M.B.London, B.S., F.R.C.S.) | 1s. 8d. |
| "The Nurse's Materia Medica" (Herbert French, M.D.) | 2s. 9d. |

Case-papers, Charts, Professional Cards, and every kind of Institutional Literature.

Of all Booksellers or of The Scientific Press, Limited, 28 and 29 Southampton Street, Strand, London, W.C.

